# Supplementary material for: Bionic 3D printed corals
Source: Nat Commun. 2020 Apr 9;11:1748. doi: 10.1038/s41467-020-15486-4 (PMC7145811; doi:10.1038/s41467-020-15486-4)
Supplement: Supplementary file 1 — Supplementary information [file 41467_2020_15486_MOESM1_ESM.pdf]

Supplementary Information for

Bionic 3D printed corals

D. Wangpraseurt et al.

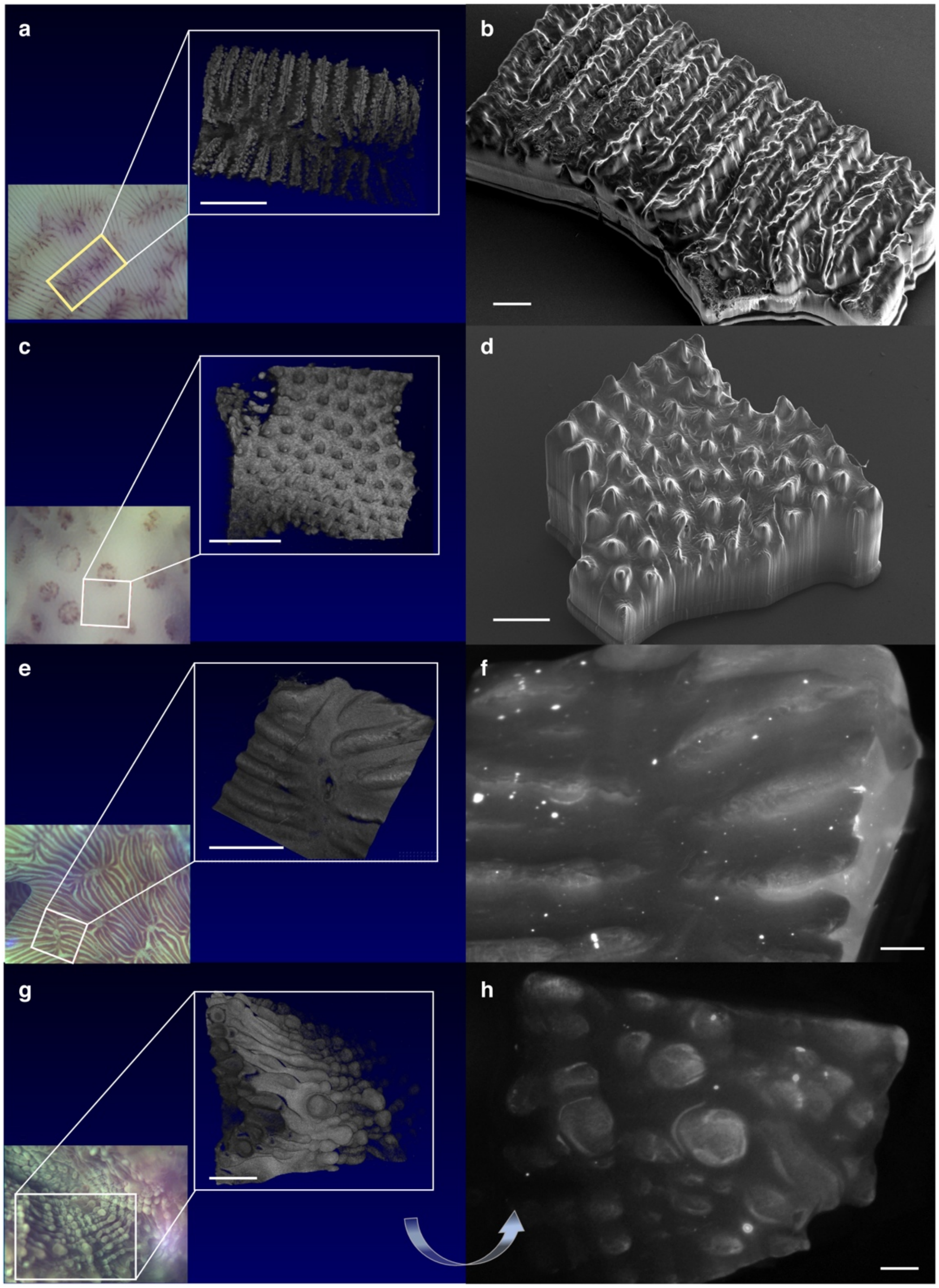

Supplementary Figure 1. Microtopography of corals and 3D bioprinted bionic corals. Skeleton of *Pavona cactus* (**a**, **b**) and *Pocillopora damicornis* (**c**, **d**) as well as tissue surface of *Pavona cactus* (**e**, **f**) and *Favites flexuosa* (**g**, **h**). USB camera images and respective optical coherence tomography scans of natural corals (**a**, **c**, **e**, **g**) and 3D printed replica (**b**, **d**, **f**, **h**). Skeletal 3D printed constructs were imaged with an environmental SEM, while 3D printed tissue constructs were photographed with a microscope camera. Scale bar = 1mm (**a**, **b**, **d**, **e**, **g**) and 500 µm (**c**, **f**, **h**).

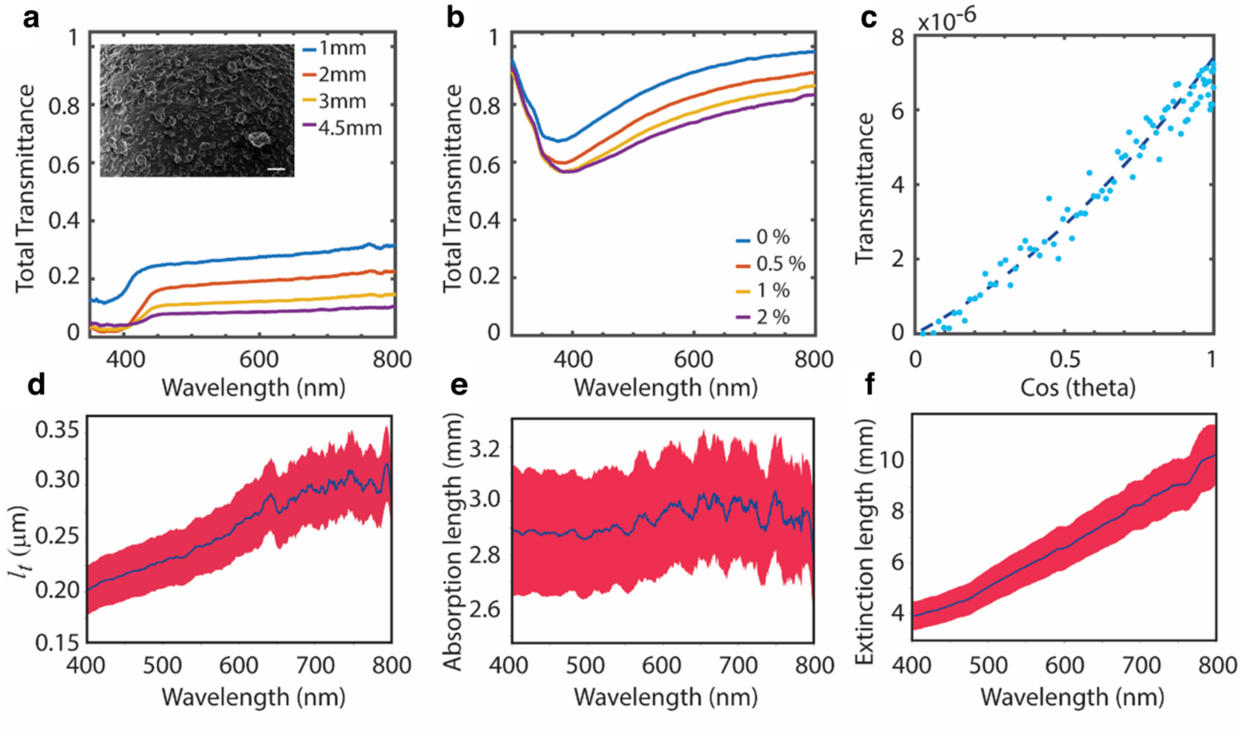

Supplementary Figure 2. **Optical characterization of 3D printed constructs.** Total transmittance of bionic skeleton with 7% CNC concentration for different slab thicknesses (1-4.5 mm) (**a**). The high CNC density yields a rough surface (see SEM image in inlet, scale bar = 40 µm). Total transmittance of bionic coral tissue doped with different concentrations of CNC (0-2%) (**b**). Fitting of extrapolation length (z_e) for bionic skeleton according to Eq. 2 based on the angular distribution of transmitted light (**c**). Calculated transport mean free path (l_t, µm) (**d**) and absorption length (l_a, mm) for bionic skeleton (mean ± CI) (**e**). Extinction length for bionic tissue estimated using Beer-Lambert law (mean ± CI) **(f).**

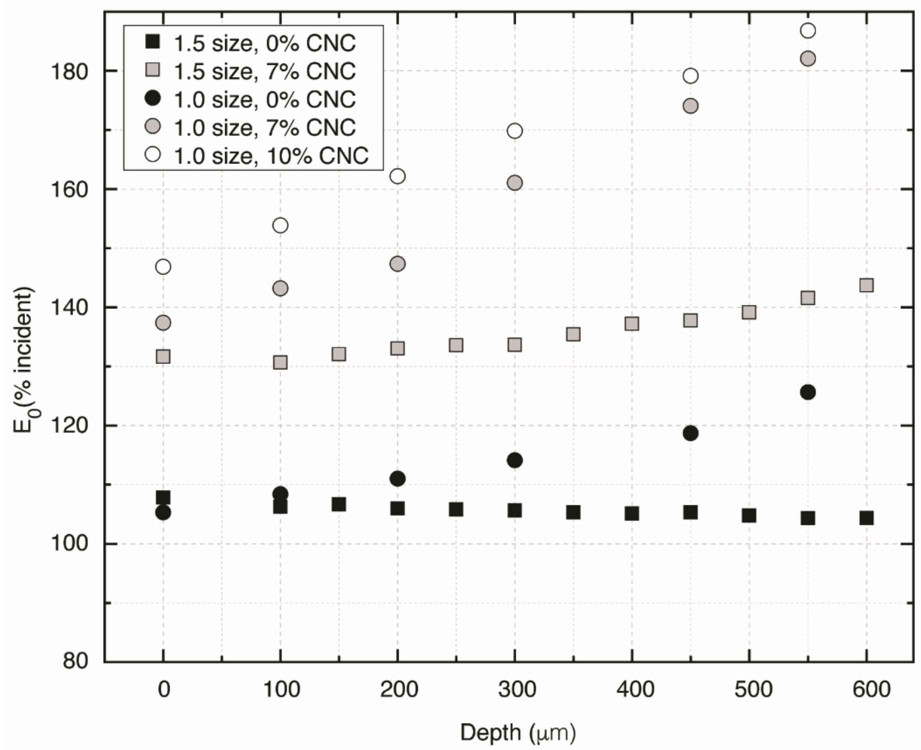

Supplementary Figure 3. Effect of CNC doping and corallite cup size on fluence rate (E_0) attenuation. Measurements were performed for different CNC concentrations (0-10%) using the original corallite cup size (maximal width = 1 mm) and a 1.5-fold enhanced size. E_0 (fluence rate) was normalized to the vertically incident downwelling irradiance E_d.

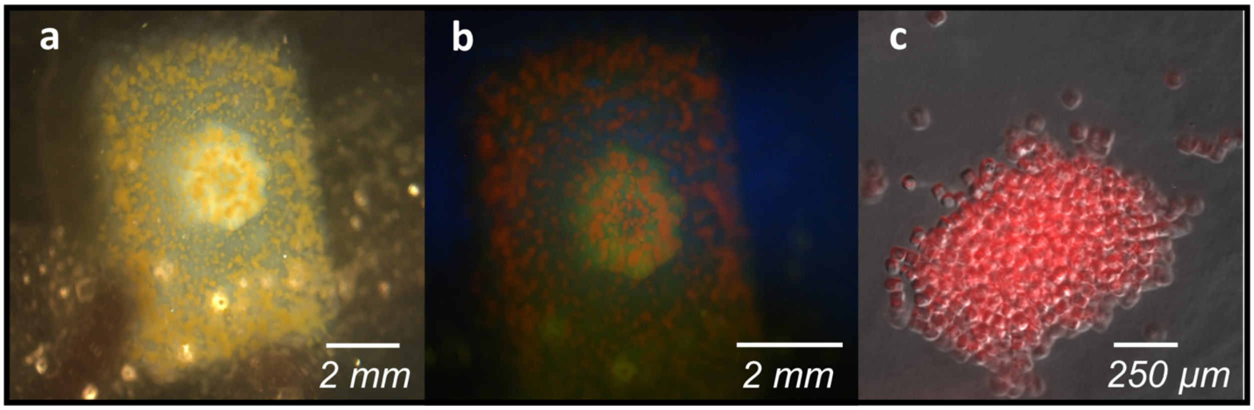

Supplementary Figure 4. ***Thalassiosira pseudonana*** **3D bioprinted culture about 7 days old**. Diatoms grow in aggregates (yellow) that are macroscopically visible (a). Chlorophyll fluorescence imaging of the same bioprint (b) and close up of an individual diatom aggregate (c).

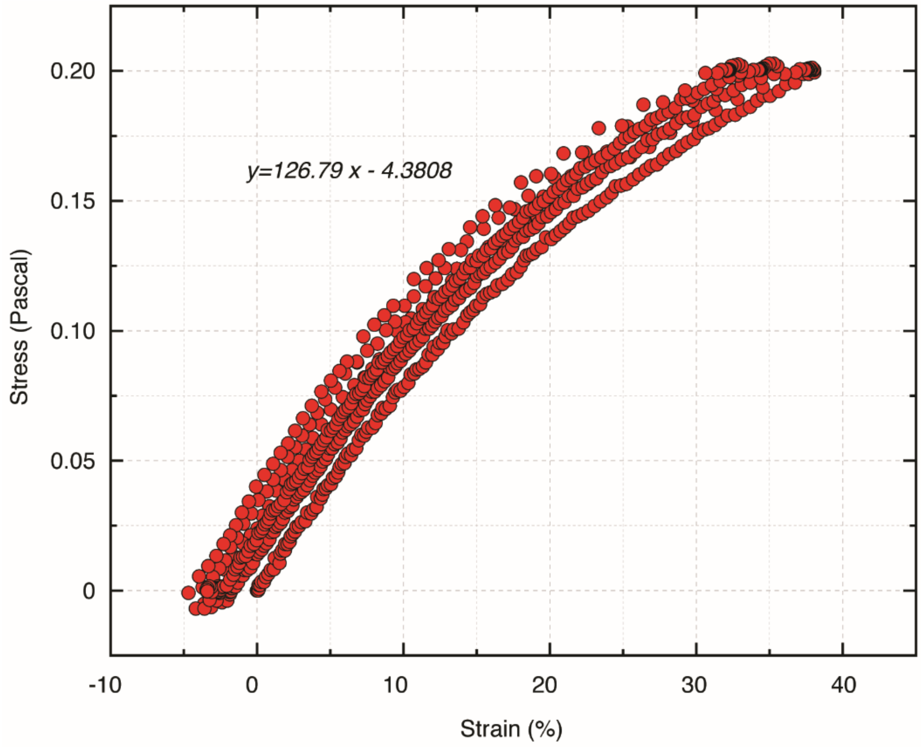

Supplementary Figure 5. Stress-strain analysis of coral-inspired bionic tissue. Replicate measurements of 6 bionic tissues were performed. The average elastic modulus was E = 4.3 kPa.
