# Supplementary material for: Bionic 3D printed corals
Source: Nat Commun. 2020 Apr 9;11:1748. doi: 10.1038/s41467-020-15486-4 (PMC7145811; doi:10.1038/s41467-020-15486-4)
Supplement: Supplementary file 3 — Description of Additional Supplementary Files [file 41467_2020_15486_MOESM3_ESM.docx]

**Description of Additional Supplementary Files**

**File name:** Supplementary data 1

**Description:** STL file of Pocillopora damicornis tissue expanded

**File name:** Supplementary data 2

**Description:** STL file of intermediate bionic skeleton

**File name:** Supplementary data 3

**Description:** STL file of final simplified skeleton

**File name:** Supplementary data 4

**Description:** STL file of final simplified tissue

**File name:** Supplementary data 5

**Description:** STL file of skeleton of Pavona cactus

**File name:** Supplementary data 6

**Description:** STL file of coenostal spines of Pocillopora damicornis

**File name:** Supplementary data 7

**Description:** STL file of tissue surface of Pavona cactus

**File name:** Supplementary data 8

**Description:** STL file of tissue surface of Favites flexuosa
