# Supplementary material for: Bionic 3D printed corals
Source: Nat Commun. 2020 Apr 9;11:1748. doi: 10.1038/s41467-020-15486-4 (PMC7145811; doi:10.1038/s41467-020-15486-4)
Supplement: Supplementary file 12 — Reporting Summary [file 41467_2020_15486_MOESM12_ESM.pdf]

nature research

Corresponding author(s): Daniel Wangpraseurt, Silvia Vignolini, Shaochen Chen

Reporting Summary

Nature Research wishes to improve the reproducibility of the work that we publish. This form provides structure for consistency and transparency in reporting. For further information on Nature Research policies, see Authors & Referees and the Editorial Policy Checklist.

Statistical parameters

When statistical analyses are reported, confirm that the following items are present in the relevant location (e.g. figure legend, table legend, main text, or Methods section).

n/a	Confirmed	
☐	☒	The exact sample size (n) for each experimental group/condition, given as a discrete number and unit of measurement
☐	☒	An indication of whether measurements were taken from distinct samples or whether the same sample was measured repeatedly
☒	☐	The statistical test(s) used AND whether they are one- or two-sided<br>*Only common tests should be described solely by name; describe more complex techniques in the Methods section.*
☒	☐	A description of all covariates tested
☒	☐	A description of any assumptions or corrections, such as tests of normality and adjustment for multiple comparisons
☐	☒	A full description of the statistics including central tendency (e.g. means) or other basic estimates (e.g. regression coefficient) AND variation (e.g. standard deviation) or associated estimates of uncertainty (e.g. confidence intervals)
☒	☐	For null hypothesis testing, the test statistic (e.g. F, t, r) with confidence intervals, effect sizes, degrees of freedom and P value noted<br>*Give P values as exact values whenever suitable.*
☒	☐	For Bayesian analysis, information on the choice of priors and Markov chain Monte Carlo settings
☒	☐	For hierarchical and complex designs, identification of the appropriate level for tests and full reporting of outcomes
☒	☐	Estimates of effect sizes (e.g. Cohen's d, Pearson's r), indicating how they were calculated
☐	☒	Clearly defined error bars<br>*State explicitly what error bars represent (e.g. SD, SE, CI)*

Our web collection on statistics for biologists may be useful.

Software and code

Policy information about availability of computer code

Data collection	Optical coherence tomography data was collected with ThorImage OCT 4.4. The bionic coral was designed in Autodesk 3ds Max 2016b.Integrating sphere data and optical goniometer data was acquired with Matlab 2016b. Oxygen microsensor data was acquired with SensorTraceSuite v3.1. (Unisense). Scalar irradiance measurementes were recorded with AvaSoft8 (Avantes). Confocal microscopy scans were acquired with NIS confocal elements (Nikon).
Data analysis	Tetrahedral mesh generation and optical modeling were performed using the open-source Matlab-based software Iso2mesh (http://iso2mesh.sourceforge.net) and mmclab (http://mcx.sf.net/cgi-bin/index.cgi?MMC/Doc/MMCLAB), respectively. Surface rendering of OCT data was performed in Matlab and Meshlab 2016. Curve fitting (cell density and photosynthesis-irradiance data) was performed in Origin Pro 2018.

For manuscripts utilizing custom algorithms or software that are central to the research but not yet described in published literature, software must be made available to editors/reviewers upon request. We strongly encourage code deposition in a community repository (e.g. GitHub). See the Nature Research guidelines for submitting code & software for further information.

Data

Policy information about availability of data

All manuscripts must include a data availability statement. This statement should provide the following information, where applicable:

- Accession codes, unique identifiers, or web links for publicly available datasets
- A list of figures that have associated raw data
- A description of any restrictions on data availability

All data are available in the main text or the supplementary materials.

Field-specific reporting

Please select the best fit for your research. If you are not sure, read the appropriate sections before making your selection.

- [x] Life sciences
- [ ] Behavioural & social sciences
- [ ] Ecological, evolutionary & environmental sciences

For a reference copy of the document with all sections, see nature.com/authors/policies/ReportingSummary-flat.pdf

Life sciences study design

All studies must disclose on these points even when the disclosure is negative.

Sample size

No sample size calculation was performed for bionic coral cultivation experiments. Initial experiments suggested that the chosen sample size was adequate based on the magnitude and consistency of microalgal cell growth over 12 days.

Data exclusions

Observations of cell viability and performance during the material optimization process is not shown, only the successful final bionic coral design. A subset (day 1 and day 12) of scalar irradiance data is shown for the cultivation experiment, while intermediate days are omitted to improve clarity.

Replication

All experimental findings were reliably reproduced.

Randomization

During bionic coral cultivation experiments, bionic prints were randomly chosen for cell count estimates as well as photosynthesis an scalar irradiance measurements. For each bionic print, photosynthesis and scalar irradiance measurements were performed on randomly chosen polyp areas.

Blinding

Investigators determined microalgal cell density blinded.

Reporting for specific materials, systems and methods

Materials & experimental systems

n/a	Involved in the study
x	Unique biological materials
x	Antibodies
x	Eukaryotic cell lines
x	Palaeontology
x	Animals and other organisms
x	Human research participants

Methods

n/a	Involved in the study
x	ChIP-seq
x	Flow cytometry
x	MRI-based neuroimaging
